# Artificially Sweetened Beverages and the Response to the Global Obesity Crisis

**DOI:** 10.1371/journal.pmed.1002195

**Published:** 2017-01-03

**Authors:** Maria Carolina Borges, Maria Laura Louzada, Thiago Hérick de Sá, Anthony A. Laverty, Diana C. Parra, Josefa Maria Fellegger Garzillo, Carlos Augusto Monteiro, Christopher Millett

**Affiliations:** 1 Post-Graduate Program in Epidemiology, Federal University of Pelotas, Pelotas, Brazil; 2 MRC Integrative Epidemiology Unit, School of Social and Community Medicine, University of Bristol, Bristol, United Kingdom; 3 Department of Nutrition, School of Public Health, University of São Paulo, São Paulo, Brazil; 4 Center for Epidemiological Studies in Health and Nutrition, University of São Paulo, São Paulo, Brazil; 5 Public Health Policy Evaluation Unit, School of Public Health, Imperial College London, London, United Kingdom; 6 Washington University in St. Louis School of Medicine, Program in Physical Therapy, St. Louis, Missouri, United States of America; 7 PhD Program on Global Health and Sustainability, School of Public Health, University of São Paulo, São Paulo, Brazil; 8 Department of Epidemiology, Institute of Social Medicine, State University of Rio de Janeiro, Rio de Janeiro, Brazil

## Abstract

Christopher Millett and colleagues argue that artificially sweetened beverages should not be promoted as part of a healthy diet.

**Summary Points**

- In March 2015, the World Health Organization (WHO) published revised guidelines on sugar intake that call on national governments to institute policies to reduce sugar intake and increase the scope for regulation of sugar-sweetened beverages (SSBs).
- In face of the growing threat of regulatory action on SSBs, transnational beverage companies are responding in multiple ways, including investing in the formulation and sales of artificially sweetened beverages (ASBs), promoted as healthier alternatives to SSBs.
- The absence of consistent evidence to support the role of ASBs in preventing weight gain and the lack of studies on other long-term effects on health strengthen the position that ASBs should not be promoted as part of a healthy diet.
- The promotion of ASBs must be discussed in a broader context of the additional potential impacts on health and the environment. In addition, a more robust evidence base, free of conflicts of interest, is needed.

## Introduction

Dietary intake of added sugars has increased dramatically worldwide during the past few decades, coinciding with increases in obesity and noncommunicable diseases. About 75% of all processed foods and beverages contain added sugar in the United States [1]. Consumption of sugar-sweetened beverages (SSBs), including carbonated soft drinks, fruit-flavored drinks, sports/energy drinks, and ready-to-drink coffees and teas, contribute to over 46% of added sugar in the US diet [2], are the second largest source in Brazilians’ diet [3], and constitute nearly a third of sugar intake among British adolescents [4]. Though SSBs are a major contributor to total calorie intake, they contain few, if any, essential nutrients. There is convincing epidemiological evidence linking SSB consumption to increased risk of overweight and obesity and type II diabetes [5–7].

In March 2015, the World Health Organization (WHO) published revised guidelines on sugar intake [8], calling on national governments to institute policies to reduce sugar intake to less than 10% of total energy. The guidance notes that additional health benefits would be derived if sugar intake could be reduced to less than 5% of total energy. These recommendations have prompted an increased focus on policy actions to reduce sugar intake worldwide; particularly, interventions to curb SSB consumption have been implemented or proposed within a small number of jurisdictions [9]. These include taxes on SSBs in Mexico [10], France [11], Hungary [12], and the US (Philadelphia [13] and Berkeley [14]) and most recently proposed in the United Kingdom [15] and Ireland [16]. Warning labels have been implemented or proposed in California [17] and Chile [18], and advertising restrictions and banning of sales in schools in Brazil [19] and Chile [18]. While evidence on the effectiveness of these interventions is still emerging, recent research has found that the introduction of an SSB tax in Mexico was associated with a 6% reduction in sales, with a larger impact in low socioeconomic status households [10].

## Response of Transnational Beverage Companies to Increasing Pressure for SSB Regulation

Transnational beverage companies recognize that addressing growing health concerns about their products is needed to guarantee share of sales, volume growth, and overall financial results. As an example, in the 2014 official annual mandatory report for the US Securities and Exchange Commission, Coca-Cola declared that “some researchers, health advocates and dietary guidelines are suggesting that consumption of sugar-sweetened beverages…is a primary cause of increased obesity rates and are encouraging consumers to reduce or eliminate consumption of such products” [20]. In the same report, it is stated that “possible new or increased taxes on sugar-sweetened beverages by government entities to reduce consumption or to raise revenue; additional governmental regulations concerning the marketing, labeling, packaging or sale of our sugar-sweetened beverages; and negative publicity resulting from actual or threatened legal actions against us or other companies in our industry relating to the marketing, labeling or sale of sugar-sweetened beverages may reduce demand for or increase the cost of our sugar-sweetened beverages, which could adversely affect our profitability” [20].

In face of the growing threat of regulatory action on SSBs, transnational beverages companies are responding in multiple ways, including lobbying for voluntary self-regulation [21], influencing scientific research [22], promoting sports and physical activity events and research as part of their social responsibility strategy [23], and contributing to election campaigns [21]. They are also targeting emerging markets (e.g., China, India, and Latin America), making substantial investments in global marketing and in the reformulation of products [24]. Within this context, artificially sweetened beverages (ASBs), such as soft drinks, flavored water, juices, and ready-to-drink tea and coffee containing artificial sweeteners, have emerged as an important alternative to maintain industries’ sales and profits. Global beverage industries have been investing in expanding their portfolios and shifting production to these products, perceived as healthier by some consumers.

ASB sales account for approximately one-quarter of the soft drink market globally [25,26]. There is evidence of a shift towards greater ASB consumption in some settings. In the US, the proportion of children consuming ASBs doubled between 1999–2000 and 2007–2008 [27,28]. In Australia, purchases of carbonated ASBs increased by 24% between 1997 and 2011 [29,30], representing an annual growth rate of 3.3% in contrast to an annual decline of 0.7% in SSBs [30]. Global sales data indicate substantial heterogeneity across regions, with SSBs predominately accounting for growth in soft drink sales in emerging economies, such as Chile, Brazil, and China [31]. However, ASB consumption may increase in these settings as concerns about growing rates of obesity influence both consumer preference and government willingness to act.

## Current Evidence Regarding the Health Impact of ASBs

ASBs are marketed as healthy alternatives to SSBs based on their characteristic of mimicking the sensory properties of SSBs while providing null (or low) energy content. The potential benefits from ASBs rely on the assumption that they elicit no (or incomplete) energy compensation. However, there are long-standing concerns that ASBs may trigger compensatory mechanisms [32,33], which could offset a reduction in energy and sugar intake provided by their replacement of SSBs. The main proposed mechanisms are that ASBs stimulate sweet taste receptors—which could theoretically increase appetite, induce preference for sweet taste, and modulate gut hormone secretion—or result in overconsumption of solid foods due to awareness of the low calorie content of ASBs [33].

Evidence of the long-term impact of ASBs on weight management and related health outcomes is limited. Systematic reviews of observational studies indicate that ASB intake is positively associated with increased body mass index in both children [34] and adults [6,35] and to cardiometabolic disease risk (e.g., type 2 diabetes [6] and stroke [36]). However, findings from observational studies might be biased by residual confounding, due to the clustering of lifestyle factors, and reverse causality, as overweight/obese people are more likely to consume ASBs in an attempt to control weight [37].

The effect of ASBs on weight management has been tested in some randomized controlled trials (RCTs). These have produced mixed findings, with some studies indicating a null effect [38–45], while others have found modest reductions in weight [43,46–51]. Most of these studies have numerous limitations, including short intervention and follow-up periods, small sample size, absence of intention-to-treat analysis, multicomponent intervention (e.g., motivational calls and visits for the ASB group only), and participant losses. A systematic review published in 2012 [35], funded by the International Life Sciences Institute (ILSI), a nongovernmental organization largely funded by the food/beverage industry [52], reported that replacement of SSBs by ASBs resulted in modest weight loss.

More recently published data suggest that artificial sweeteners may contribute to the development of glucose intolerance by altering the composition and functions of gut microbiota [53]. Observational prospective studies suggest that ASB intake is positively related to glucose levels, although these results might be influenced by publication bias and residual confounding [6,35]. Few RCTs have been conducted in the general population, and the results are inconclusive [54].

An important consideration when interpreting these findings is the potential for conflicts of interest, as industry-sponsored research is common [55]. Systematic reviews sponsored by food or beverage companies or with other conflicts of interest are more likely to report a conclusion of no positive association between SSB consumption and obesity than those reporting having no industry sponsorship or conflicts of interest [55]. Recently, a systematic review found that ASB industry-sponsored reviews were also more likely to report favorable results and conclusions regarding ASB effects on weight control than nonsponsored reviews. The opposite occurred with reviews sponsored by competitor food companies (e.g., the sugar industry), which were more likely to report negative results compared to nonsponsored reviews. Importantly, the authors estimated that “almost half of the reviews had authors that failed to disclose relevant conflicts of interest with the food industry” [56]. These findings underscore the substantial and complex nature of bias from conflicts of interest in ASB research.

In summary, the available evidence does not directly support a role of ASBs in inducing weight gain or metabolic abnormalities but also does not consistently demonstrate that ASBs are effective for weight loss or preventing metabolic abnormalities. Evidence on the impact of ASBs on child health is even more limited and inconclusive than in adults.

## Environmental Impacts of Sweetened Beverages

The environmental consequences of ASBs and SSBs should also be closely scrutinized given their negligible nutritional benefits and potential detrimental health impacts. High consumption of sweetened beverages leads to high generation of solid waste and cumulative chemical pollution, affecting marine life and contaminating the food chain, which raise concerns regarding food safety for human health [57–59]. The volume of water required to produce a 0.5 L plastic bottle of carbonated soft drink is estimated to range from 150 up to 300 L of water [60]. In the UK, the consumption of soft drinks in 2011 (14.685 billion L) contributed about 4.5 million tonnes CO_2_e in the atmosphere [61], or approximately 300 g of CO_2_e per L. In addition, artificial sweeteners have been recently recognized as an emerging environmental contaminant of the aquatic environment. Households, small business enterprises, and industry contribute to releasing sweeteners into the aquatic environment, with scarce research on their ecotoxicological profile and consequences for planetary health [62,63].

## Implications for Policy

Although current guidelines developed for public health authorities and consumers consistently recommend that SSB consumption should be discouraged [8,64–66], they provide contrasting recommendations regarding ASB intake. As an example, the American Heart Association (AHA) and the American Diabetes Association (ADA) guidance states that replacing sugar for artificial sweeteners in foods and drinks may result in modest weight loss, although it is acknowledged that supporting evidence may be insufficient [66], while the Pan-American Health Organization (PAHO) indicates that both beverages with excessive free sugar (≥10% of total energy) and beverages with any amount of other sweeteners (“food additives that impart a sweet taste to a food”) should be subjected to regulatory measures [64,67].

Overall, there is consensus across national food-based dietary guidelines, which recommend avoiding or reducing SSB intake. However, guidance on ASB consumption is mixed (see S1 Table for a list of guidelines) [68]. Guidelines that discourage ASB intake for the general population (e.g., Australia, Brazil, Bulgaria, Guatemala, Honduras, Italy, Panama, Qatar, and Switzerland) argue that ASBs are not adequate sources of hydration, may not help in weight reduction/control, or may increase the risk of dental erosion due to acidity levels (in the case of carbonated beverages). A few guidelines (e.g., France, Georgia, Turkey, and the US) recommend ASBs as an alternative to SSBs for weight control, but with several caveats, such as stating that water is always the best choice and that ASBs may not be an effective long-term weight management strategy.

Few food policies targeting SSBs also address ASBs. In Mexico, the soft drink tax only covered SSBs (excluding ASBs and alcoholic and dairy beverages) [10], while the French soda tax covers both SSBs and ASBs [69]. The Brazilian school feeding program guidelines forbid the acquisition of any soft drinks [19], while the US Department of Agriculture (USDA) Smart Snacks in School standards allow the availability of low-calorie flavored and/or carbonated beverages for high school students [70]. On 16 June 2016, Philadelphia’s city council voted to pass a tax of 1.5 cents per ounce on both SSBs and ASBs. To what degree SSB-focused policies encourage the consumption of ASBs is not yet clear. Recent experiences from California and Mexico suggest that taxing SSBs led to a partial substitution of SSBs with nontaxed beverages, mainly bottled water [10,71].

Caution should be exercised in engaging transnational beverage companies in future research. The US National Caries Program (NCP) was launched in 1971 with an aim of preventing tooth decay. There is evidence that the sugar industry acted to influence research priorities, and several strategies were adopted that focused on reducing the harms of sugar consumption rather than restricting intake [72].

Given the lack of consistent data on the effectiveness of ASBs on weight control, the potential impact of bias from industry sponsorship in ASB research, the high generation of solid waste and water use, and the largely unknown long-term impacts of ASBs on human health and on the environment, we argue that there are sufficient grounds to advise against policies that directly or indirectly promote their consumption. In practice, this means that ASBs should not be recommended in dietary guidance and be subject to the same restrictions on advertising and promotion as those imposed on SSBs. New taxes implemented on SSBs should be applied at the same level to ASBs. The impact of these policies on SSB and ASB consumption, as well as health and environmental outcomes, should be subject to rigorous and independent evaluation.

## Conclusion

The absence of evidence to support the role of ASBs in preventing weight gain and the lack of studies on other long-term effects on health strengthen the position that ASBs should not be promoted as part of a healthy diet. The promotion of ASBs must be discussed in a broader context of the additional potential impacts on health and the environment. In addition, a more robust evidence base, free of conflicts of interest, is needed. Far from helping to solve the global obesity crisis, characteristics related to ASB composition (low nutrient density and food additives), consumption patterns (potential promotion of sweet taste preference), and environmental impact (misuse of natural resources, pollution, or ecotoxicity) make them a potential risk factor for highly prevalent chronic diseases.

## Supporting Information

S1 Table List of food-based dietary guidelines published after the year 2000 in countries with a prevalence of obesity higher than 15%. (DOCX)

## Abbreviations

ADA: American Diabetes Association
AHA: American Heart Association
ASB: artificially sweetened beverage
ILSI: International Life Sciences Institute
NCP: National Caries Program
PAHO: Pan-American Health Organization
RCT: randomized controlled trial
SSB: sugar-sweetened beverage
USDA: US Department of Agriculture
WHO: World Health Organization
