# Supplementary material for: Artificially Sweetened Beverages and the Response to the Global Obesity Crisis
Source: PLoS Med. 2017 Jan 3;14(1):e1002195. doi: 10.1371/journal.pmed.1002195 (PMC5207632; doi:10.1371/journal.pmed.1002195)
Supplement: S1 Table — (DOCX) [file pmed.1002195.s001.docx]

**S1 Table.** List of food-based dietary guidelines published after the year 2000 in countries with prevalence of obesity higher than 15%

| **Countries** | **Prevalence of Obesity^1^ (%)** | **Food-based dietary guidelines^2^** | |
| --- | --- | --- | --- |
|  |  | Links | Year (Language) |
| Albania | 17.6 [12.7-22.2] | [FBDG of ALB](http://www.fao.org/3/a-as658e.pdf) | 2008 (E) |
| Antigua e Barbuda | 30.9 [24-38] | [FBDG of AT](http://www.fao.org/3/a-as848e.pdf)G | 2007 (F) |
| Argentina | 26.3 [21.3-31.4] | [FBDG of ARG](https://www.assal.gov.ar/assa/documentacion/guias_alimentarias.pdf) | 2003 (S) |
| Australia* | 28.6 [24-32.9] | [FBDG of AUS](http://www.nhmrc.gov.au/guidelines-publications/n55) | 2013 (E) |
| Bahamas | 36.2 [29.9-42.4] | [FBDG of BHS](http://www.fao.org/3/a-as849e.pdf) | 2002 (E) |
| Barbados | 31.3 [25-37.9] | [FBDG of BRB](http://www.fao.org/3/a-as851e.pdf) | 2009 (E) |
| Belgium | 20.2 [15.3-24.4] | [FBDG of BEL](http://www.fao.org/3/a-as664f.pdf) | 2005 (F) |
| Belize | 22.5 [17.4-27.8] | [FBDG of BLZ](http://www.fao.org/3/a-as852e.pdf) | 2012 (E) |
| Brazil* | 20 [15.8-24] | [FBDG of BRA](http://www.fao.org/nutrition/education/food-dietary-guidelines/regions/countries/brazil/en/) | 2014 (P) |
| Bulgaria* | 23.2 [17.6-28.5] | [FBDG of BGR](http://ncphp.government.bg/files/hranene-en.pdf) | 2006 (E) |
| Canada | 28 [23.9-32.6] | [FBDG of CAN](http://www.hc-sc.gc.ca/fn-an/alt_formats/fnihb-dgspni/pdf/pubs/fnim-pnim/2007_fnim-pnim_food-guide-aliment-eng.pdf) | 2007 (E) |
| Chile | 27.8 [ 22.8-32.7] | [FBDG of CHL](http://www.inta.cl/Consumidores/Revistas/guia_de_alimentacion.pdf) | 2013 (S) |
| Costa Rica | 24.3 [19.9-28.4] | [FBDG of CRC](http://www.fao.org/3/a-as859s.pdf) | 2010 (S) |
| Cuba | 25.2 [19.4-30.3] | [FBDG of CUB](http://www.fao.org/3/a-as864s.pdf) | 2009 (S) |
| Dominica | 25.8 [20.5-31.1] | [FBDG of DMA](http://www.fao.org/3/a-as853e.pdf) | 2007 (E) |
| Dominican Republic | 23.9 [18.6-29.2] | [FBDG of DOM](http://www.fao.org/3/a-as866s.pdf) | 2009 (S) |
| El Salvador | 21.8 [17.4-26.5] | [FBDG of SLV](http://www.fao.org/3/a-as867s.pdf) | 2012 (S) |
| Fiji | 36.4 [31.2-41.8] | [FBDG of FIJ](http://www.fao.org/3/a-as883e.pdf) | 2013 (E) |
| France‡ | 23.9 [18.9-29.1] | [FBDG of FRA](https://www.ac-strasbourg.fr/fileadmin/pro/Actions_educatives/Caaps/Pro/ens/la_sante_vient_en_mangeant.pdf) | 2002 (F) |
| Georgia‡ | 20.8 [16.2-26] | [FBDG of GEO](http://www.fao.org/3/a-as682e.pdf) | 2005 (E) |
| Gredana | 26.2 [20.8-32] | [FBDG of GRD](http://www.fao.org/3/a-as854e.pdf) | 2006 (E) |
| Guatemala* | 18.6 [14-22.9] | [FBDG of GTM](http://www.fao.org/3/a-as870s.pdf) | 2012 (S) |
| Guyana | 22.9 [17.7-28.3] | [FBDG of GUY](http://www.fao.org/3/a-as856e.pdf) | 2004 (E) |
| Honduras* | 18.2 [14.1-22.2] | [FBDG of HND](http://www.fao.org/nutrition/education/food-dietary-guidelines/regions/countries/honduras/en/) | 2012 (S) |
| Ireland | 25.6 [21.2-30.2] | [FBDG of IRL](https://www.healthpromotion.ie/hp-files/docs/HPM00796.pdf) | 2012 (E) |
| Israel | 25.3 [20.1-30.3] | [FBDG of ISR](http://www.fao.org/3/a-as685e.pdf) | 2008 (E) |
| Italy* | 21 [17-24.9] | [FBDG of ITA](http://www.fao.org/3/a-as686o.pdf) | 2003 (I) |
| Jamaica | 27.2 [21.9-32.7] | [FBDG of JAM](http://www.fao.org/3/a-az914e.pdf) | 2015 (E) |
| Lebanon | 31.9 [27.2-37.4] | [FBDG of LBN](http://www.fao.org/3/a-az914e.pdf) | 2013 (E) |
| Mexico | 28.1 [23.4-32.5] | [FBDG of MEX](http://www.imss.gob.mx/sites/all/statics/salud/guia-alimentos.pdf) | 2010 (S) |
| Namibia | 18.9 [14.4-23.5] | [FBDG of NAM](http://www.fao.org/3/a-as839e.pdf) | 2000 (E) |
| New Zealand | 29.2 [25.2-33.1] | [FBDG of NZL](http://www.health.govt.nz/our-work/eating-and-activity-guidelines/current-food-and-nutrition-guidelines) | 2008 (E) |
| Nicaragua | 17.1 [12.6-21.4] | [FBDG of NIC](https://prezi.com/xh4wxtems_zg/guia-alimentaria-de-nicaragua/) | 2012 (S) |
| Oman | 30.9 [25.5-36.4] | [FBDG of OMN](http://www.fao.org/3/a-as845e.pdf) | 2009 (E) |
| Panama* | 26.8 [21.5-31.7] | [FBDG of PAN](http://www.fao.org/3/a-as876s.pdf) | 2013 (S) |
| Paraguay | 16.3 [11.8-20.4] | [FBDG of PRY](http://www.fao.org/3/a-ax401s.pdf) | 2013 (S) |
| Portugal | 20.1 [14.8-25.3] | [FBDG of PRT](http://www.fao.org/3/a-ax433o.pdf) | 2003 (P) |
| Qatar* | 42.3 [35.9-48.4] | [FBDG of QAT](http://www.fao.org/3/a-az908e.pdf) | 2015 (E) |

| **Countries** | **Prevalence of Obesity^1^ (%)** | **Food-based dietary guidelines^2^** | |
| --- | --- | --- | --- |
|  |  | Links | Year (Language) |
| Saint Kitts and Nevis | 28.3 [22.5-34.5] | [FBDG of KNA](http://www.fao.org/3/a-as858e.pdf) | 2010 (E) |
| Saint Lucia | 26.9 [21.4-33] | [FBDG of LCA](http://www.fao.org/3/a-as860e.pdf) | 2007 (E) |
| Saint Vincent and the Grenadines | 24.3 [18.7-29.3] | FBDG [of VCT](http://www.fao.org/3/a-as861e.pdf) | 2006 (E) |
| Saudi Arabia | 34.7 [29.6-40.1] | [FBDG of SAL](http://www.moh.gov.sa/en/Ministry/MediaCenter/Publications/Documents/final%20english%20%20%D8%A7%D9%84%D9%83%D8%AA%D8%A7%D8%A8%20%D8%A7%D9%84%D8%B9%D9%84%D9%85%D9%8A%20%D8%A5%D9%86%D8%AC%D9%84%D9%8A%D8%B2%D9%8A.pdf) | 2012 (E) |
| Seychelles | 26.3 [20.5-31.7] | [FBDG of SYC](http://www.fao.org/nutrition/education/food-based-dietary-guidelines/regions/countries/seychelles/en/) | 2006 (E) |
| South Africa | 26.8 [22.8-31.3] | [FBDG of ZAF](http://www.fao.org/3/a-as842e.pdf) | 2013 (E) |
| Spain | 23.7 [19.5-28.1] | [FBDG of ESP](http://www.naos.aesan.msps.es/naos/ficheros/investigacion/Come_sano_y_muevete.pdf) | 2008 (S) |
| Sweden | 20.5 [16.3-25.2] | [FBDG of SWE](http://www.fao.org/3/a-az854e.pdf) | 2015 (E) |
| Switzerland* | 19.4 [15.6-23.5] | [FBDG of CHE](http://www.sge-ssn.ch/fr/toi-et-moi/boire-et-manger/equilibre-alimentaire/pyramide-alimentaire-suisse/) | 2011 (E) |
| Turkey‡ | 29.5 [25.2-33.8] | [FBDG of TUR](http://www.fao.org/3/a-as697e.pdf) | 2014 (E) |
| United Kingdom | 28.1 [24.5-31.8] | [FBDG of UK](http://www.fao.org/3/a-as838e.pdf) | 2007 (E) |
| USA‡ | 33.7 [29.6-37.7] | [FBDG of USA](http://health.gov/dietaryguidelines/2015/guidelines/) | 2015 (E) |
| Uruguay | 26.7 [20.8-32.1] | [FBDG of URY](http://www2.msp.gub.uy/andocasociado.aspx?5683,21582) | 2005 (S) |

Food-based dietary guidelines published - in English (E), French (F), Italian (I), Portuguese (P) or Spanish (S), after the year 2000 in countries with prevalence of obesity**^1^** higher than 15% were analyzed. Few food guidelines express recommendations regarding artificial sweeteners. Guidelines that mention potential detrimental effects of artificial sweeteners include those identified by “*” and guidelines that mention artificial sweeteners can be a potential alternative to added sugars include those identified by “‡”. Source for Prevalence of Obesity: WHO - Global Health Observatory; ages 18+; year 2014; both sexes (<http://gamapserver.who.int/gho/interactive_charts/ncd/risk_factors/obesity/atlas.html>).

**^1^** Body mass Index ≥ 30kg/m^2^

**^2^** Documents accessed in 5 September 2016.
